# Multinuclear Magnetic Resonance Spectroscopy at Ultra-High-Field: Assessing Human Cerebral Metabolism in Healthy and Diseased States

**DOI:** 10.3390/metabo13040577

**Published:** 2023-04-19

**Authors:** Pandichelvam Veeraiah, Jacobus F. A. Jansen

**Affiliations:** 1 Scannexus (Ultra-High-Field MRI Center), 6229 EV Maastricht, The Netherlands; 2 Faculty of Health Medicine and Life Sciences, Maastricht University, 6229 ER Maastricht, The Netherlands; 3 Department of Radiology and Nuclear Medicine, Maastricht University Medical Center, 6229 HX Maastricht, The Netherlands; 4 School for Mental Health and Neuroscience, Maastricht University, 6229 ER Maastricht, The Netherlands; 5 Department of Electrical Engineering, Eindhoven University of Technology, 5612 AZ Eindhoven, The Netherlands

**Keywords:** MRS, UHF, cerebral metabolism, X-nuclei MRS, multinuclear MRS, 7T, 9.4T

## Abstract

The brain is a highly energetic organ. Although the brain can consume metabolic substrates, such as lactate, glycogen, and ketone bodies, the energy metabolism in a healthy adult brain mainly relies on glucose provided via blood. The cerebral metabolism of glucose produces energy and a wide variety of intermediate metabolites. Since cerebral metabolic alterations have been repeatedly implicated in several brain disorders, understanding changes in metabolite levels and corresponding cell-specific neurotransmitter fluxes through different substrate utilization may highlight the underlying mechanisms that can be exploited to diagnose or treat various brain disorders. Magnetic resonance spectroscopy (MRS) is a noninvasive tool to measure tissue metabolism in vivo. ^1^H-MRS is widely applied in research at clinical field strengths (≤3T) to measure mostly high abundant metabolites. In addition, X-nuclei MRS including, ^13^C, ^2^H, ^17^O, and ^31^P, are also very promising. Exploiting the higher sensitivity at ultra-high-field (>4T; UHF) strengths enables obtaining unique insights into different aspects of the substrate metabolism towards measuring cell-specific metabolic fluxes in vivo. This review provides an overview about the potential role of multinuclear MRS (^1^H, ^13^C, ^2^H, ^17^O, and ^31^P) at UHF to assess the cerebral metabolism and the metabolic insights obtained by applying these techniques in both healthy and diseased states.

## 1. Introduction

The brain is the most complex and metabolically active of all organs in the human body. In a healthy adult brain, the cerebral metabolism relies mainly on glucose as a main obligatory energy substrate to support the oxygen metabolism as well as to generate biochemical energy in the form of adenosine triphosphate (ATP). Although the brain comprises only 2% of the body’s mass, it consumes 20% of the total body’s oxygen and 25% of its glucose intake at resting state. Thus, the brain consumes a large amount of energy, of which 75% is utilized for the metabolic processes related to signaling (neurotransmission) and the remaining 25% serving to maintain basic cellular functions [1]. As the brain has minimal reserves of energy storage, it requires a continuous supply of oxygen and metabolic substrates from the blood for normal brain functioning. While neurons are the major energy-consuming cells (70–80%), glial cells (astrocytes, oligodendrocytes, and microglia) play an important role in maintaining energy homeostasis by utilizing the remaining energy of 20–30% [2,3].

The cerebral metabolism of glucose is not only involved in producing the bulk amount of ATP but also in producing a wide variety of intermediate metabolites. Each metabolite can provide distinct metabolic information, as they are associated with substrates (e.g., lactate, pyruvate, glycogen), energy compounds [4,5] (e.g., creatine, Cre; phosphocreatine, PCr; nicotinamide adenine dinucleotide, NAD), neurotransmission [6,7] (e.g., glutamate, Glu; glutamine, Gln and GABA), membrane turnover [4,5] (e.g., choline-containing compounds, CCC), antioxidants [8] (e.g., glutathione, GSH; ascorbic acid, Asc), and neuroprotection [4,5,9] (e.g., N-acetyl aspartate, NAA; taurine, Tau). Moreover, some metabolites are more specific to certain cell types, such as neuronal (e.g., NAA, N-acetylaspartyl glutamate, NAAG, and Glu) and glia-related metabolites (e.g., myo-inositol, MI; choline, Cho) [4,5]. Indeed, an aberrant level of brain metabolites has widely been accepted as a surrogate biomarker for different pathological processes such as tissue necrosis (lactate), neuronal damage (NAA) [10,11], loss of membrane integrity [4,5] (CCC), astrogliosis [12] (MI), demyelination (phosphorylethanolamine, PE), mitochondrial dysfunction (Cre, ATP, PCr), and neuromodulation (NAAG) [4,5,10,12]. Besides alterations in regional metabolite level, the rate of cerebral metabolism (i.e., metabolic flux) on a cellular level is also important, as it provides novel insights towards understanding the mechanisms involved in various brain disorders [13,14,15]. Moreover, cerebral metabolic measurements may provide a reliable measure of brain function, as the cerebral metabolism is coupled to the brain activity [6,7]. Growing evidence indicates that a variety of neurological, neurodegenerative, and psychiatric brain disorders intimately share common cerebral metabolic alterations, which facilitate or trigger the progression of brain disorders. Hypometabolism (decreased glucose metabolism), impaired mitochondrial function, neuroinflammation, astrogliosis, increased reactive oxygen species (ROS) production, altered neurotransmission, and neuronal cell death are common metabolic abnormalities that have been reported in several brain disorders [16].

Despite the role of the cerebral metabolism and its alterations being noted in brain disorders, the field has been stymied by the lack of appropriate noninvasive techniques that can measure cerebral metabolism accurately and reliably with sufficient spatial, temporal, and cell-specific resolution. Positron emission tomography (PET), in combination with radioisotopes, is a commonly used metabolic imaging technique in the clinic for cerebral metabolic measurements and can provide regional/tissue metabolic changes [17,18] towards the diagnosis and monitoring of disease progression. However, PET is limited to providing information about subsequent downstream intermediate metabolites, cell-specific neuroenergetics, and corresponding metabolic fluxes. Alternatively, magnetic resonance spectroscopy (MRS) is a gold-standard technique with no risk of radiation to measure biochemicals/metabolites noninvasively and to study the cerebral metabolism in vivo [4,19,20]. Over the past two decades, standard proton (^1^H) MRS has been widely applied in research at clinical field strengths (≤3T) and can reliably quantify highly abundant metabolites such as total NAA, creatine, taurine, and myoinositol, but lacks sensitivity to detect physiologically low-abundant metabolites such as beta-hydroxybutyrate (BHB), Asc, NAD, serine, GSH, and GABA [21]. In addition to protons, a wide variety of biochemical elements, such as oxygen, deuterium, carbon, and phosphorus, play a vital role in metabolic processes. In principle, those elements with nuclei of odd spin, such as ^13^C, ^2^H, ^17^O, and ^31^P, are NMR-visible that can be detected by employing the X-nuclei MRS, which can provide valuable metabolic information (e.g., metabolic fluxes) in studying various neurological disorders. However, X-nuclei measurements generally suffers from a low signal-to-noise ratio (SNR), which is primarily due to the inherent low NMR sensitivity and low biodistribution.

Nowadays, a recent transition towards ultra-high-field (UHF, >4T) has led to significant improvements to study the cerebral metabolism in more detail due to a linear gain in SNR and a high spectral resolution that enables the detection of physiologically low-abundant metabolites as well as to perform X-nuclei MRS. UHF-MRS is promising, as it provides a more sensitive tool to monitor the aberrant metabolism than at clinical field strength (≤3T) [22]. Certain MRS methods can be employed based on hyperpolarization and chemical exchange saturation transfer (CEST) to study the brain metabolism, but those methods are not covered in this review. The aim of this review is to provide a brief overview of current MRS techniques, including both proton (^1^H) and X-nuclei (e.g., ^13^C, ^2^H, ^17^O, and ^31^P) applications at UHF, to assess the cerebral metabolism in humans. In addition, we will demonstrate briefly how emerging/advanced MRS modalities at UHF, such as functional MRS and diffusion-weighted MRS, have the potential to determine the dynamic changes of the metabolites level and cell-specific metabolic interactions involved in neurodegenerative diseases, respectively. Finally, a comprehensive overview of the cerebral metabolism obtained by these techniques at UHF in both healthy and diseased states during the past two decades is provided.

### 1.1. Multinuclear MR Spectroscopy as a Noninvasive Tool to Measure Cerebral Metabolism

Currently, advances in medical imaging not only allow the observation of structural changes but also the assessment of pathophysiological and metabolic changes towards achieving more precise personalized medicine for various brain disorders. MR modalities are a well-established, noninvasive diagnostic tool that play a vital role in biomedical research. A nucleus with an odd spin number (I) can be MR-detectable, such as ^13^C, ^2^H, ^17^O, and ^31^P. The ability to acquire an adequate signal for such nuclei detection depends on several factors, including natural abundance, NMR sensitivity (which depends on gyromagnetic ratio, nuclear spin number, and field strength), and relaxation times (T_1_ and T_2_), as shown in Table 1.

Protons (^1^H) are the most preferred nuclei for MR due to their high abundance in the human body and strong MR sensitivity. A standard proton MRI has great strength to reveal anatomical information by depicting a variety of image contrasts, such as *T*_1_, *T*_2_, proton density, diffusion, susceptibility, chemical exchange, magnetization transfer, and perfusion, while MRS offers relevant biochemical information such as the tissue viability or the metabolic state of a tissue. By combining both MRI and MRS techniques, the metabolism of a specific location of the tissue can be assessed. Both modalities share the common underlying concepts of MR physics, though MRS exploits differences in resonance frequencies (chemical shift) between signals from protons with different chemical surroundings. For instance, when a volunteer is subjected to an external strong magnetic field (B0), the net magnetization of MR active nuclei (e.g., proton) in the body will process at a particular frequency that is referred to as the Larmor frequency. A very small difference/shift in such Larmor frequency can occur depending on the chemical group of molecules situated, a phenomenon that is called electronic shielding. Thus, resonance frequencies can vary for different molecules within the nucleus. For example, proton molecules bound in fat and water resonate at different frequencies, giving rise to fat and water peaks separately in two different positions in the MR spectrum. The area under the peak is proportional to the number of detectable molecules within the nuclei, which makes MRS a quantitative technique. In addition, the amplitude and linewidth of the peak depends on the relaxation times (T_1_ and T_2_) of the metabolite (e.g., fat) detected within the nuclei. For instance, the long T_2_ metabolites appear as a narrower peak than the short T_2_ metabolites.

Standard ^1^H-MRS allows for the estimation of absolute (or relative) concentrations of many endogenous metabolites (Table 1) in a particular region/tissue in the brain. Moreover, ^1^H-MRS can provide information about how fast the production and depletion of such metabolites, especially for lactate and glucose, is happening in the target tissue/region (i.e., metabolic rate). In addition to the regional/tissue metabolic changes, cell-specific metabolites (neuron and glia-related) and corresponding metabolic fluxes can be monitored in a dynamic manner using exogenous isotopically labeled substrates by employing ^2^H and ^13^C-MRS. Interestingly, ^31^P-MRS allows the detection of endogenous molecules and can provide information about energy metabolites (e.g., PCr, ATP) and membrane-related metabolites such as phosphomonoesters (PME) and phosphodiesters (PDE). Due to its noninvasive and dynamic nature, multinuclear MRS is ideally suited for in vivo studies to study the cerebral metabolism, as it enables the measurement of total metabolite concentration, metabolic fluxes, the neurotransmission rate, and the monitoring of cerebral metabolic changes longitudinally in response to an intervention.

Depending on the localization of the MRS signal, MRS can be broadly divided into two categories: Firstly, single voxel spectroscopy (SVS), which employs a small and single volume of interest (VOI), referred as a voxel, in the targeted tissue to obtain a high quality of the MR spectrum with short scan time, which provides an accurate measure of the total concentration of metabolites in the selected region. Secondly, CSI/MRSI (chemical shift imaging/magnetic resonance spectroscopic imaging), which employs multiple voxels in a large VOI, providing a specific metabolite map in the form of an MR image throughout the region/organ. Thus, MRSI is used to obtain the spatial distribution of metabolites in one-, two-, or three-dimensional directions, and therefore leads to longer scan times when compared to SVS. Both proton and X-nuclei measurements can be applied in those two techniques, MRS and MRSI. The detailed MR physics concepts and methodological aspects can be found in textbooks [27] and review articles [28,29], as it is beyond the scope of this review.

### 1.2. Relevant Metabolic Substrates for Cerebral Metabolism

The brain derives most of its energy from the blood by obtaining oxygen and metabolic substrates via the blood–brain barrier (BBB). Cellular constituents of the BBB are mainly astrocytes and pericytes, which possess substrate-specific transporters that act as gatekeepers for the entry of metabolic substrates into the brain. Although glucose is the preferred and main obligatory substrate for a healthy adult brain, other substrates such as lactate, glycogen, short-chain fatty acids (e.g., acetate), ketone bodies (e.g., beta-hydroxy butyrate), and amino acids (e.g., Glu, Gln, and GABA) can be used to sustain the cerebral metabolism for a short moment during certain anaerobic and hypoglycemic conditions [30]. The uptake of such metabolic substrates into the brain is influenced by several factors, including the cellular composition of the target region, the availability of circulating substrates, the distribution of substrate-specific transporters, enzymes, and neuronal activity. Therefore, the type and amount of substrate utilization are known to vary in different brain regions in both healthy and diseased states [16]. The common metabolic substrates and their relevant characteristic features are summarized in Table 2.

### 1.3. Metabolic Pathways and Relevant MRS Techniques to Assess Cerebral Metabolism

**Figure 1:**
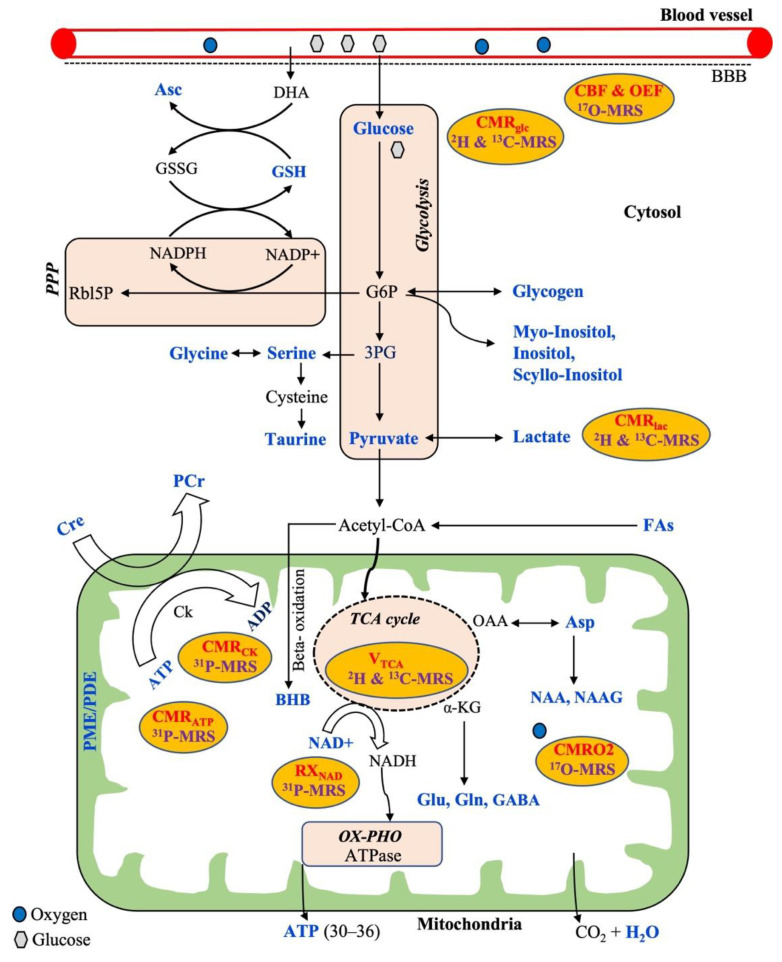
Overview of a neural cell showing common metabolic pathways (brown) for energy production with relevant metabolic substrates, metabolic intermediaries, and corresponding measurable metabolic parameters (in red) which are related to the respective multinuclear MRS techniques (purple). All the metabolites highlighted in a blue color are possible to detect by MRS techniques. *Abbreviations:* PPP, pentose phosphate pathway; OX-PHO, oxidative phosphorylation; TCA cycle, tricarboxylic acid cycle; G6P, glucose-6-phosphate; 3PG, 3-phosphoglycerate; BBB, blood–brain barrier; DHA, dehydroascorbate; Rbl5P, ribulose-5-phosphate; GSH, glutathione; GSSG, oxidized glutathione; Asc, ascorbic acid; NAD, nicotinamide adenine dinucleotide; BHB, beta-hydroxybutyrate; Cre, creatine; PCr, phosphocreatine; Ck, creatine kinase; ATP, adenosine triphosphate; ADP, adenosine diphosphate; FAs, fatty acids; Asp, aspartate; NAA, N-acetyl aspartate; NAAG, N-acetylasparatate glutamate; Glu, glutamate; Gln, glutamine; GABA, gamma aminobutyric acid; α-KG, alpha ketoglutarate; PME, phosphomonoesters; PDE, phosphodiesters; CMR_glc_, CMR_lac_, CMRO2, CMR_CK,_ CMR_ATP_—cerebral metabolic rate of glucose, lactate, oxygen, creatine kinase, and ATP, respectively; CBF, cerebral blood flow; OEF, oxygen extraction fraction; RX_NAD_, redox rate of NAD.

The general metabolic pathways available for energy production are glycolysis, the tricarboxylic acid cycle (TCA cycle), the pentose phosphate pathway (PPP), and oxidative phosphorylation (OX-PHO), as shown in Figure 1. Glycolysis is the first pathway of energy production that occurs in all brain cells, where glucose is converted into pyruvate through ten enzyme-catalyzed reactions. In the first step of glycolysis, glucose-6-phosphate (G6P) is produced. Although the majority of G6P is degraded into pyruvate, it also acts as a precursor for the synthesis of phospholipids (myoinositol, inositol, and scyllo-inositol) and glycogen that occur predominantly in astrocytes rather than neurons. Alternatively, glucose can be oxidized through a second pathway referred to as the pentose phosphate pathway (PPP) via G6P that produces NADPH and 5-carbon carbohydrates (e.g., Rbl5P), which are necessary for the regeneration of antioxidants (e.g., GSH) and nucleic acid synthesis, respectively. The synthesized NADPH is used to maintain GSH in a reduced state for cellular protection against reactive oxygen species (ROS). Additionally, ascorbic acid (Asc) is regenerated from the external source of dehydroascorbate (DHA, the oxidized form of Asc) at the expense of reduced GSH. Moreover, the nonessential amino acids, such as glycine, serine, and taurine, can be synthesized from the 3-phosphoglycerate (3-PG), an intermediate product of glycolysis. The glucose-derived pyruvate can have two fates: either to form lactate in the cytosol or to enter the third pathway of the mitochondrial TCA cycle through forming acetyl-CoA. The pyruvate-derived acetyl CoA in mitochondria not only produces ATP but also several intermediaries to synthesize important neurotransmitters (Glu, Gln, Asp, GABA, NAA, NAAG). In addition, acetyl-CoA can be derived from free fatty acids via the BBB that can undergo mitochondrial beta-oxidation to produce ketone bodies including beta-hydroxybutyrate (BHB) that occurs mostly in astrocytes.

**Figure 2:**
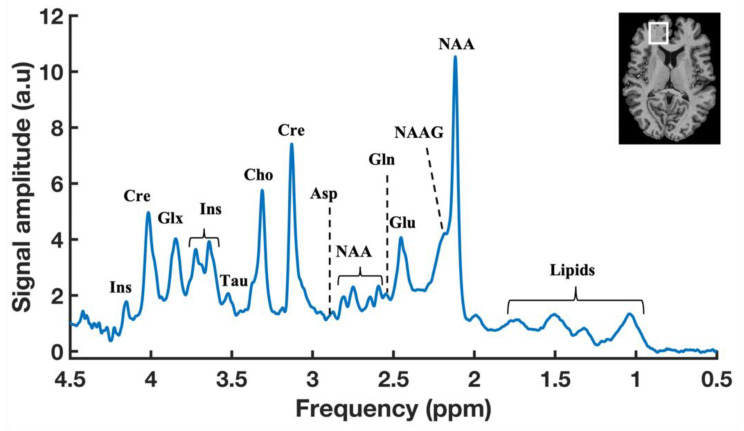
A typical ^1^H-MR brain spectrum obtained by SVS with a localization scheme of using Semi-Localized by Adiabatic SElective Refocusing (sLASER) sequence (repetition time (TR) = 7000 ms, echo time (TE) = 30 ms, signal averages = 60, voxel = 25 × 20 × 17 mm, and acquisition time = 7 min) in the prefrontal cortex (PFC) of a healthy volunteer at 7T. Anatomical MR image of human brain showing the representation of voxel position placed in the PFC region (Top right).

In addition, oxygen is required from the blood flow for the complete oxidation of glucose via the above-mentioned metabolic pathways towards producing the end-products of CO_2_, ATP, and water (Figure 1). Therefore, the cerebral metabolic rate (CMR) is assessed by measuring mainly three parameters: the amount of oxygen, glucose utilization, and blood flow into the brain. Thus, CMR is represented by the measurements of the cerebral blood flow (CBF), the cerebral metabolic rate of glucose (CMR_glu_), oxygen extraction fraction (OEF), and the cerebral metabolic rate of oxygen (CMRO2) in the brain. Generally, proton MRS can detect a wide variety of intermediate metabolites that occur from the substrate metabolism, as shown in the typical ^1^H-MR brain spectrum obtained by SVS (Figure 2). ^17^O MRS imaging techniques are used to measure the cerebral metabolic rate of oxygen (CMRO2), oxygen extraction fraction (OEF), and cerebral blood flow (CBF) [54,55]. In addition to CMR_glc_ and CMR_lac_, mitochondrial TCA fluxes can be measured non-invasively by applying ^2^H and ^13^C-MRS with the use of an isotope-labeled substrate [15,56,57]. ^31^P-MRS provides information about energy metabolites (e.g., Cre, PCr, ATP), membrane integrity (e.g., PME/PDE ratio), enzyme kinetic information (e.g., CMR_ck_), and metabolic fluxes associated with ATP (e.g., CMR_ATP_). In addition, ^31^P-MRS can provide NAD concentrations, which is a vital coenzyme involved in cerebral energy metabolism [58,59]. Moreover, the ratio of [NAD+]/[NADH] is termed as the redox ratio, which reflects the intracellular redox state that can be measured by ^31^P-MRS [59,60].

## 2. Advantages of UHF-MRS to Assess Cerebral Metabolism

UHF-MR scanners can provide not only detailed anatomical and functional information in the human brain, but also allow the assessment of valuable metabolic information of a cell/tissue. Nowadays, there are more than 70 7T MR scanners available worldwide being applied for human research. Very few research centers have 9.4T MR scanners, and only one 11.7T scanner is available for human brain research [61].

Increased SNR at UHF has enabled to perform single voxel MRS in deeply embedded structures of the human brain, such as the pons [62], thalamus [21], hippocampus [63,64], putamen [62,65], precentral gyrus [66], basal ganglia [63], corpus callosum [67], nucleus accumbens [68], striatum [69], centrum semiovale [70], cerebellar vermis [62], and substantial nigra [62]. Moreover, UHF-MRS can theoretically incorporate shorter scan times due to a high SNR when compared to lower field strength [21], although, in practice, scan times often remain the same with better spectral quality. In addition, the high SNR achieved at UHF enables to quantify the physiologically low-abundant metabolites, such as ascorbic acid [71], GSH [72], NAD [58], serine [73], 2-hydroxyglutrate (HG) [74,75], GABA [21,76], and glycine [77], which is generally quite challenging at a lower field strength.

In addition to a high SNR, UHF-MRS provides better spectral resolution when compared to lower field strengths. This is the most noteworthy aspect of UHF usage for MRS applications, as it enables to distinguish many overlapped metabolites. For example, glutamate and glutamine are commonly quantified as a glx measure at clinical field strengths, but these two metabolites can be resolved clearly at UHF, which has been demonstrated in studies comparing 7T with 3T [78,79,80,81] and a high field strength, 4T [22]. Another interesting common metabolite, namely NAA, can be distinguished well from the NAAG [82,83], a ubiquitous molecule which has a quite distinct function, commonly quantified as total NAA (tNAA) in earlier MRS studies at a lower field strength. Moreover, a few studies demonstrated the feasibility of quantifying overlapped low-abundant metabolites such as GSH [84] and GABA [85,86] with standard MRS techniques at UHF without the requirement of sophisticated spectral editing techniques.

It is widely known that relaxation times depend on various factors, including the type of nuclei detection, target tissue, metabolite, and field strength. Altered relaxation times at UHF permit to detect specific metabolites. For example, long-T_2_ metabolites can be detected at UHF without much SNR loss. Similarly, short T_1_ metabolites can be averaged more to achieve an adequate SNR. Taken together, UHF-MRS provides a reliable and reproducible measure of neurochemical profiles in different target regions of the human brain. Indeed the potential of UHF-MRS has been demonstrated in a multicenter study at 7T in the human brain using Semi-Localized by Adiabatic SElective Refocusing (sLASER) [87] and by using the STimulated Echo Acquisition Mode (STEAM) sequence [62]. Moreover, UHF is highly beneficial for MRSI applications, as it provides a high SNR that allows us to achieve a reliable metabolite map throughout the brain in a short scan time [88,89], which was demonstrated in comparison with 3T and 7T [90].

As stated earlier, X-nuclei MRS at a lower field strength is quite challenging due to the inherent low NMR sensitivity and low biodistribution (Table 1). For example, the concentration of brain glycogen detected by ^13^C-MRS is <1 millimolar, which is approximately 20 times lower than the proton-observed metabolites such as NAA detected by ^1^H-MRS. In addition, the low intrinsic MR sensitivity of X-nuclei hinders the accurate and reliable detection of such low-abundant X-nuclei metabolites due to the inherent low SNR, particularly at a lower field strength (<3T). Most importantly, X-nuclei biomedical applications, such as ^13^C, ^31^P, ^17^O, and ^2^H, will be highly benefited with the use of UHF-MRS. The signal enhancement and spectral improvement of UHF, especially for X-nuclei applications, has been nicely illustrated in a review by Zhu et al. [91].

## 3. Proton MRS at UHF

### 3.1. Spectral Editing ^1^H-MRS Techniques to Detect Metabolic Substrates

Proton MRS detects a large number of brain metabolites based on its chemical shift information [92]. In a typical ^1^H-MR brain spectrum with SVS, more than 20 metabolites can be detected, as shown in Figure 2, but still some metabolites, including brain substrates such as glucose, BHB, and lactate, are not visualized as distinct peaks in the standard ^1^H-MR spectrum. First, the physiological concentrations of those substrates are very low (Table 2). Secondly, those substrates are generally overlapped with other metabolites, mainly the huge lipid and/or water resonances, and, therefore, they are not visible with standard ^1^H-MRS techniques. It is possible to detect those substrates by advanced MRS techniques using spectral editing ^1^H-MRS techniques. Such spectral editing techniques can be employed by using different approaches, such as J-difference editing, 2D-resolved, long-TE, T_1_-based editing, coherence transfer, and multiple-quantum filter (MQF) [93,94]. All these approaches aim to reduce/minimize the unwanted resonances, such as lipid and/or water contamination, towards identifying the small resonance of the overlapped target metabolites. The methodological approaches and recommendations for spectral editing techniques are provided in a publication by the MRS consensus group [93]. At a lower field strength, such spectral editing techniques are necessary, and the most common application is GABA spectroscopy in the brain.

In addition to those metabolic substrates, spectral editing methods have been applied at 7T to edit a large number of overlapped metabolites of our interest, such as ascorbic acid (vitamin C) [95,96], 2-hydroxyglutarte (HG) [74,75], GABA [76], NAAG [97,98], NAD [58], glycine [77,99], and serine [73] in the human brain. With a high SNR and improved spectral resolution at UHF, the editing efficiency can be improved, and thereby UHF-MRS can provide a reliable measure of overlapped metabolites [100].

#### 3.1.1. Detection of Glucose

Glucose has a complex spectral pattern arising from 14 protons in two isoforms, such as alpha and beta glucose (with an approximate 36:64 ratio in in vivo). Alpha glucose resonance occurs at 5.23 ppm, beta glucose at 4.63, 3.23 ppm, and combination of both at 3.8 ppm [27]. As brain glucose is in very low concentrations, glucose infusions are conventionally used to detect brain glucose by ^1^H-MRS. In addition to the low concentrations of glucose, all the glucose resonances are completely overlapped with various metabolites, such as choline (3.20 ppm), methine (5.13 ppm), lipid (1.30 ppm), and water peaks, indicating the challenges to resolve glucose peaks with standard ^1^H-MRS. The alpha glucose at 5.23 ppm is partially separate from other peaks but it is present only 50% of beta glucose. Gruetter et al. demonstrated the in vivo feasibility of alpha glucose detection at 4T in the human brain with standard short TE ^1^H-MRS, but it requires excellent water suppression, as the huge water (4.70 ppm) peak is relatively close to the alpha glucose. Alternatively, MQF approaches can be used to detect both alpha and beta glucose, but this approach inherently leads to 50% signal loss. Recently, Kaier et al. applied the J-difference editing (JDE) method at 7T [101] and demonstrated the in vivo quantification of the intrinsic brain glucose level by detecting beta glucose resonance at 3.23 ppm. This method provides better sensitivity at UHF and does not require any ^13^C-labeled glucose.

#### 3.1.2. Detection of Lactate

In addition to the low concentrations of brain lactate, the target lactate resonance at 1.33 ppm is partially overlapped with lipids (1.30 ppm) and macromolecules (1.2 ppm). However, it is possible to detect lactate with conventional short TE protocols at UHF, but it requires a sophisticated postprocessing pipeline to account for macromolecular and lipid contamination [38]. Alternatively, long TE ^1^H-MRS was used for the detection of brain lactate at 7T using the semiLASER sequence with a TE of 144 ms [102]. Although long TE is useful for minimizing lipid contamination, it leads to T_2_-associated signal loss in lactate detection when compared to short TE protocols. Lactate measurements with three protocols, such as short TE (16 ms), long TE = 110 ms, and short TE with presuppressing lipids with an inversion time (TI) of 300 ms, were compared in a study at 7T. The protocols with short TE and prelipid suppression with short TE showed reliable and reproducible measure of lactate in the human brain at 7T [103]. Moreover, the JDE method was applied in a few studies at 7T to detect lactate without the contamination of macromolecules [100,104].

#### 3.1.3. Detection of Beta-Hydroxybutyrate (BHB)

BHB has six nonexchangeable protons, and the corresponding peaks appear at four different positions, such as methyl protons at 1.19 ppm, methylene protons at 2.29 and 2.39 ppm, and methine protons at 4.13 ppm in the MR spectrum [27]. Among them, the most common target for BHB detection is the doublet peak at 1.19 ppm, which is completely overlapped with a huge lipid resonance at 1.30 ppm, as well as lactate (1.33 pm) and macromolecules (1.2 ppm) which hampers the accurate detection of BHB with standard short TE ^1^H-MRS. Therefore, spectral editing methods are necessary to edit the BHB peak from the unwanted lipid resonances. With the JDE method, at an optimum TE, the methine resonance at 4.13 ppm will be targeted by an editing pulse to detect the BHB peak at 1.19 ppm in the edited spectrum. Although BHB was detected by using JDE-^1^H-MRS in the human brain at 4T [51,52], so far there are no studies performed at 7T. On the other hand, the potential benefits of ketone bodies have been evaluated. Recently, a ^1^H-MRS study at 7T showed that Glu and GABA levels were reduced by the acute ingestion of ketone supplements [105].

## 4. X-Nuclei MRS Applications at UHF

### 4.1. ^13^C-MRS to Determine Cellular Metabolic Fluxes and Neurotransmitter Cycling Rate

**Figure 3:**
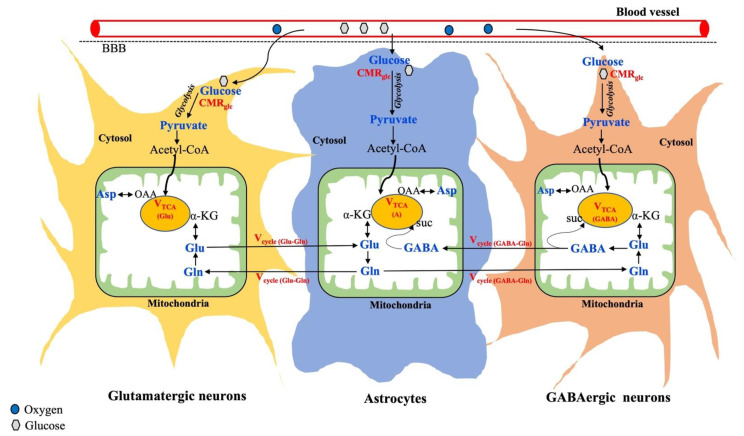
Overview of metabolic interactions between neurons (glutamatergic and GABAergic) and astrocytes. Metabolic fluxes/cycles that can be measured by ^13^C-MRS are highlighted in red color. Suc, Succinate; OAA, Oxaloacetate. Adapted from the publication [106], with permission.

The cerebral metabolism in the brain is known to be compartmentalized between neurons and glia, as shown in Figure 3.

Further, neurons are broadly classified into two categories based on neuronal activity: Glutamatergic neurons are the most abundant (80%) and involved in excitatory activity. GABAergic neurons are less abundant (20%) and responsible for inhibition [57]. As shown in Figure 3, glucose is taken up by both neurons and astrocytes, where it gets metabolized into pyruvate through glycolysis. In all brain cells, the pyruvate-derived acetyl-CoA enters the mitochondrial TCA cycle, where alpha-ketoglutarate is synthesized, which acts as an important precursor for the synthesis of glutamate. The synthesized glutamate produces GABA only in the GABAergic neurons and glutamine is exclusively produced in astrocytes. Thus, astrocytes serve as the energetic demands of glutamatergic activity and maintain neuroenergetics through the recycling of Glu, Gln, and GABA (Figure 3). In addition to regional variation in total metabolite concentration, understanding changes in the cell-specific metabolite level and corresponding neurotransmitter fluxes through different substrate utilization may highlight the underlying mechanisms involved in various brain disorders.

^13^C-MRS was developed about 30 years ago, and since then, various developments, including RF coil design, MR sequences, signal enhancement approaches, infusion protocol, quantitative analysis, and metabolic modeling, have been improved to yield meaningful results despite its low NMR sensitivity (gyromagnetic ratio = 10.71 MHz/T) and low natural abundance (1.1%) [107,108]. Due to the large ^13^C-chemical shift range (>200 ppm), ^13^C-MRS is considered an ideal technique to detect several physiologically low-abundant metabolites without any ambiguity. The natural abundance of brain glucose detection with ^13^C-MRS is not feasible since glucose is in very low concentrations in the brain [109]. Alternatively, with exogenous ^13^C-labeled glucose infusions, brain glucose concentrations can be reliably measured by ^13^C-MRS. In addition, specific ^13^C carbon-labeled exogenous substrates, such as glucose and acetate, can be used to track the neuronal and astroglia metabolism, respectively. Thus, the total V_TCA_ cycle can be further delineated into the neuronal (V_TCA,N_) and astrocytic TCA cycle (V_TCA,A_). Moreover, the neuronal TCA cycle can be distinguished into two compartments by tracking ^13^C-incorporated cell-specific metabolites, such as glutamate and GABA, which allows us to determine the corresponding glutamatergic (V_TCA(Glu)_) and GABAergic TCA cycle (V_TCA(GABA)_) fluxes. In addition, neuroenergetics associated with the astrocyte metabolism can be studied in detail by tracking neurotransmitter cycles via the glutamate–glutamine cycle (V_cycle,Glu/Gln_) and GABA–glutamine (V_cycle,GABA/Gln_), as shown in Figure 3. All of this can be studied by ^13^C-MRS and, indeed, the application of ^13^C-MRS has been well demonstrated in both animals [110,111,112] and humans [109,113]. Relevant methodological and detailed procedures can be found elsewhere [57]. Thus, ^13^C-MRS in combination with specific ^13^C-labeled substrates not only measures the total concentration of metabolites, but also provides valuable information about metabolic fluxes, corresponding neurotransmission rate, and cell-specific interactions between neurons and glia.

Although several signal enhancement approaches, such as the nuclear overhauser effect (NOE), decoupling strategies, hyperpolarization, and indirect ^13^C MR techniques, have been employed, the widespread use of ^13^C-MRS in humans at UHF is limited. This is mainly due to the fact that ^13^C-MRS requires expensive ^13^C-labeled substrates (e.g., 1-^13^C glucose can cost ~350 $/g, Cambridge Isotope Laboratories Inc., Tewksbury, MA, USA), specific RF coil availability, and the complexity involved in ^13^C MR acquisition (e.g., SAR limitation) and analysis. So far, only two ^13^C-MRS studies have been performed in the human brain at 7T [114,115]. In one study, nonlocalized ^13^C-MRS was performed in the occipital cortex after infusion of uniformly labeled ^13^C-glucose and the TCA cycle rate determined at steady state using bicarbonate as a marker [115]. In another study, signal enhancement approaches such as nuclear overhauser enhancement (NOE) and proton decoupling were applied with ^13^C-MRS together with 2-^13^C-labeled glucose and the metabolic turnover of Glu, Gln, and Asp monitored in the visual cortex [114]. In addition, one preliminary study at 7T [116] showed the in vivo feasibility of detecting the natural abundance of neurotransmitters (Glu, Gln, and Asp) in the human brain at 7T. To partly overcome the challenges of ^13^C-MRS, Boumezbeur et al. developed an indirect ^13^C-MRS technique to detect ^13^C-incorporated metabolites based on the heteronuclear scalar coupling of ^1^H and ^13^C with the use of standard short-TE ^1^H-MRS without a ^13^C RF coil. Recently the application of this technique has been shown at 9.4T to determine mitochondrial metabolic fluxes in the human brain with oral administration of ^13^C-labeled glucose [117].

#### ^13^C-MRS to Detect Brain Glycogen

Apart from the glucose metabolism, ^13^C-MRS can further assess the role of other metabolic substrates such as lactate, glycogen, ketone bodies, and acetate in cerebral neuroenergetics in both healthy and diseased states. For example, the natural abundance of the glycogen signal (100.5 ppm) can readily be detected by direct ^13^C-MRS in the liver and muscle, even without the enrichment of ^13^C-labeled glucose, because these organs have high glycogen concentrations. A ^13^C-MRS study at 7T showed in vivo feasibility of quantifying the natural abundance of glycogen content in the human skeletal muscle [118]. As the brain has a very low glycogen content than in the muscle and liver, the infusion of ^13^C-labeled glucose is necessary together with ^13^C-MRS to study the brain glycogen metabolism. Moreover, the infusion of ^13^C-labeled glucose is required for a long time to reach a steady-state level towards quantifying the cerebral glycogen content accurately because the complete turnover of brain glycogen pool is estimated to take 3–5 days [43]. Considering these challenges, a very few ^13^C-MRS studies quantified the glycogen level in the human brain with the infusion of ^13^C-labeled glucose at 4T [43,44,119]. Among them, one study by Oz et al. attempted to measure brain glycogen accurately by infusing labeled glucose for about 80 h on five healthy volunteers and the estimated glycogen was found to be 7.8 µmol/g [43]. To date, glycogen detection at 7T in the human brain has not been reported.

### 4.2. ^2^H-MRS to Measure Global Glycolytic and Mitochondrial Metabolic Fluxes

The use of deuterium in metabolic studies has been recognized for over a century, but its potential was not realized well. Recently, deuterium MRS has gained a large interest in the MR research community and has been considered as a simple, safe, and robust method to study the in vivo metabolism with a high spatial and/or temporal resolution [56,120]. ^2^H-MRS enables to monitor a wide range of deuterated substrates, including glucose and acetate, and their metabolic products, such as water, lactate, and glx measure (Glu and Gln), in the brain. ^2^H-MRS provides additional detail of downstream metabolites compared to analogous methods such as fluorodeoxyglucose (FDG)–PET and can be easily implemented on clinical MR systems. In addition, the ^2^H-MRS technique has several advantages over ^13^C-MRS and could be used as an alternative method for the noninvasive detection of metabolic pathways and fluxes in vivo. First, the deuterated substrates are not so costly as ^13^C-labeled substrates (1-^13^C glucose can cost ~350 $/g while 6,6 2H_2_-glucose can cost ~160 $/g, Cambridge Isotopes Laboratories Inc), and it is completely safe to use for either oral or intravenous administration in human volunteers. Since deuterium-labeled substrates are nontoxic, the usage of deuterium has been well demonstrated without any side effects in both animal [121,122] and human studies [123,124,125]. The short T_1_ relaxation time of deuterium (as shown in Table 1) enables to acquire more averages for gaining SNR that increases the temporal resolution. As deuterium has very low natural abundance (0.016%), it only provides deuterated metabolites in the spectrum, and it does not require any lipid and water suppression techniques which are commonly required for standard ^1^H-MRS. Taken together, the low resonance frequency of deuterium even at UHF makes it ideal for UHF applications, as it is less susceptible for B0 inhomogeneities and chemical shift displacement (CSDE) effects. Indeed, the potential of UHF for ^2^H-MRSI has been demonstrated at 7T [123,124] and 9.4T [125] in humans to measure metabolic fluxes with good spatial and temporal resolution. Recently, de Graaf et al. showed that ^2^H-MRS has twice as high sensitivity when compared to ^13^C-MRS [124]. However, it is important to note that ^2^H-labeled glycogen was barely detectable with ^2^H-MRS due to very short T_2_ (<2 ms), leaving ^13^C-MRS as the preferred technique for in vivo glycogen detection [126]. Moreover, ^2^H-MRS enables to measure glycolytic fluxes in the cytosol and mitochondrial total V_TCA_ cycle, but it is limited to provide the corresponding neurotransmission rate in a cellular level. This is mainly due to low resolution and large linewidth, as it is difficult to distinguish deuterated form of glutamate and glutamine in the spectrum. Therefore, the Glu-Gln cycle and GABA-Gln cycle cannot be studied well by ^2^H-MRS, but it is possible with ^13^C-MRS, as explained above [15,120]. The detailed methodological approaches were covered in relevant reviews [15,120]. Alternatively, Rich et al. [127] introduced a new technique to monitor the deuterium labeling of metabolites by measuring the signal reduction in ^1^H-MRS after the administration of deuterium-labeled substrates [128,129]. It is known that deuterated substrates enable a reduction in signal loss in protons as it replaces proton molecules with deuterium labeling. Therefore, the incorporation of deuterated metabolites can be monitored indirectly by the signal loss in protons while using standard ^1^H-MRS without the requirement of a special RF coil. Recently, Henning et al. demonstrated the in vivo feasibility of ^2^H-MRS in both direct and indirect detection at 9.4T in humans [130].

### 4.3. ^17^O-MRS to Quantify Cerebral Oxygen Consumption and Blood Flow

^17^O is a stable isotope, MR-detectable nucleus with very low natural abundance of 0.037% and a low gyromagnetic ratio, as shown in Table 1. In addition, the relaxation properties of ^17^O are distinct from other nuclei, as it is independent of magnetic field strength (B0) [131]. The relaxation properties of ^17^O water are very short (<7 ms) [25], which enables to perform more averages at UHF for gaining SNR within short scan time. Moreover, the inherent linear gain in SNR at UHF makes it even possible to obtain the 3D-MRSI of ^17^O tissue (approximately 20 mm) in the human or animal brain with adequate SNR [91]. Although ^17^O_2_ MRS and ^15^O_2_-PET techniques share the common principle in determining CMRO2 using the inhalation of isotope-labeled oxygen gas, ^17^O_2_-MRS specifically detects only the metabolically generated water as other ^17^-O molecules either bound to hemoglobin or dissolved in the blood are not MR visible to the ^17^O detection [54,55]. Generally, ^17^O_2_ MRS imaging techniques are used to measure the cerebral metabolic rate of oxygen (CMRO2), cerebral blood flow (CBF), and oxygen extraction fraction (OEF). Those methodological approaches and the importance of ^17^O-MRS in the cerebral metabolism are explained in detail elsewhere, or see the relevant review by Zhu et al. [54,55]. Interestingly, the ^17^O-based CMRO2 approach enables to perform multiple CMRO2 measurements with excellent reproducibility, as metabolized ^17^O water signal can quickly reach a steady state level (<10 min in rodents) after the inhalation of oxygen gas [132]. Although the oxygen metabolism is explored well in animal studies, only a few limited ^17^O-MRS studies have been performed in humans at UHF due to several reasons, including a large body size and slow gas-exchange process in humans, [133,134]. In addition, this technique requires an expensive ^17^O-labeled water (20% of ^17^O water can cost ~750 $/g, Cambridge Isotope Laboratories Inc.), a specific RF coil, sophisticated postprocessing pipelines, and a mathematical model for accurate quantification. A ^17^O-MRI study at 7T revealed that CMRO2 was higher in normal-appearing gray matter (NAGM) than normal-appearing white matter (NAWM) in both healthy and glioma patients. On the other hand, CMRO2 measured in the tumor region was more reduced than in gray matter (GM) and white matter (WM), indicating that oxidative glycolysis was reduced in patients with glioma [134].

### 4.4. ^31^P-MRS to Assess Energy Metabolism and Membrane Integrity

^31^P-MRS is a powerful noninvasive tool to study the energy metabolism without the requirement of isotope-labeled substrates, since the ^31^P-natural abundance is high. ^31^P-MRS provides valuable information about energy metabolites, membrane integrity, enzyme kinetics, the metabolic fluxes of ATP, and physiologically relevant parameters, such as intracellular pH and the Mg^2+^ concentration in the brain. The methodological approaches of ^31^P-MRS are explained in detail elsewhere, or see the relevant review by Zhu et al. [54,55,135]. In contrast to protons, ^31^P relaxation times have shown to be decreased with increasing field strengths [60], and this enables to acquire more averages for gaining SNR at UHF. As ^31^P-metabolites have very short relaxation times (T_2_), it requires fast (e.g., ultra-short TE) MR protocols/sequences. Therefore, ^31^P-MRS at UHF is mostly performed with MRSI rather than single voxel MR spectroscopy. Indeed, the high SNR and improved spectral resolution at UHF enable to perform ^31^P-MRSI in three dimensions covering the entire human brain, and the sensitivity was compared between 4T and 7T in the visual cortex [60]. So far, seven ^31^P-MRS studies [14,59,60,136,137,138,139] have been performed in the human brain at 7T and three studies at 9.4T [88,140,141]. A few UHF-^31^P MRS studies (together with magnetization transfer) have shown the feasibility to assess the creatine kinase rate (CMR_ck_) [14,136,137,138] and ATP synthesis rate (CMR_ATP_) [136,137,138] in the human brain of healthy volunteers. Among them, one study at 7T even showed the in vivo feasibility to assess CMR_ck_ and CMR_ATP_ in both gray matter (GM) and white matter (WM). Interestingly, the measured metabolic flux of ATP was shown almost three times higher in GM, while the Ck rate was seven times higher in GM than WM, indicating that 77% of energy consumption occurs in the GM of human brain [138]. Moreover, the same study reported for the first time that the human cortex consumes an average of approximately 4.7 billion molecules of ATP per second at rest, indicating that brain is a highly energetic organ [138]. With the use of UHF, the intracellular concentrations of NAD+ and the redox ratio in the human brain of healthy volunteers was determined for the first time by Zhu et al. [59]. After this initial report, nowadays, a few studies at 7T have demonstrated the in vivo feasibility of quantifying cerebral NAD in the human brain by using both ^1^H and ^31^P-MRS methods [58]. In order to further increase SNR and to perform localized ^31^P-MRS, an indirect ^31^P-MRS method, proton-observed phosphorus editing (POPE), was developed and showed potential applications in tumor imaging at 7T [139]. At 9.4T, a high resolution pH map in the human brain of both healthy and tumor patients has been shown by ^31^P-3D-MRSI [88]. Recently a single voxel ^31^P-MRS study performed at 9.4T in the human brain compared four different MR sequences. Interestingly, the study concluded that ISIS, image selected in vivo spectroscopy, sequence provides high SNR for all ^31^P-metabolites with good localization accuracy and better spectral resolution by distinguishing even the small resonance of both intra- and extracellular inorganic phosphate (Pi) [141], indicating the potential of UHF to study cerebral energetics.

## 5. Advanced MRS Techniques

### 5.1. Functional MR Spectroscopy to Determine Dynamic Changes of Metabolites

The brain performs continuous cognitive and sensory processes throughout our life, and thus the brain is not really at rest. Every action that we do in our daily life occurs with a continuous exchange of information among neural cells via electrical synapses using biochemicals (neurotransmitters). For example, when we speak, the speech areas and networks of the human brain are activated. Over 120 years ago, such brain activation was demonstrated with an increase in CBF, which is accompanied by an increase in glucose utilization. In addition, other substrates and/or metabolite levels can be altered during brain activation, as neuronal metabolism is coupled to the blood flow. While functional MRI (fMRI) localizes the activated brain areas during specific task, functional MRS (^1^H-fMRS) might yield a more direct measure of behaviorally relevant neural activity (both excitatory and inhibitory) and synaptic plasticity in the activated brain region [142]. In 1991, Prichard et al. demonstrated the potential of ^1^H-MRS at 2.1T to measure dynamic changes of the lactate level in response to brain activity and showed increased lactate level upon prolonged visual stimulation [143]. Since this initial report, the increment of the lactate level in response to brain activity has been largely confirmed by various research groups with different stimuli responses, including visual, auditory, and panic disorders [34,144,145]. In 2005, fMRS was extended to measure dynamic changes in the level of neurotransmitters, specifically increased Glu and Gln, in response to acute pain [146], and the Glu increase upon neural activation was further confirmed by a few more studies [144,147]. In addition, beta-hydroxybutyrate (BHB) was detected by fMRS using sophisticated spectral editing sequences at 4T [51]. Nowadays, UHF-MRS has boosted the fMRS applications and generated more interest in measuring task-induced changes of physiologically low-abundant metabolites (e.g., GABA, aspartate) and substrates (e.g., BHB and glucose) related to neuroenegertics and neurotransmission [148]. In line with this, several research groups reported small changes in the concentration of lactate [34,144], BHB [51], glutamate [144,147], glucose, and aspartate in the human cortex during prolonged stimuli [148] and during transcranial direct current stimulation (tDCS) [149,150].

### 5.2. Diffusion Weighted MR Spectroscopy to Identify Different Cell Types in the Brain

The cerebral metabolism and its function results from the coordination of different cell types in the brain. It is widely accepted that brain cells can be distinguished indirectly, as a few metabolites are more specific to certain cell types. For example, neurons are expected to have high concentrations of NAA while astrocytes have high myoinositol concentrations. Moreover, certain substrates, such as acetate, fatty acids, and glycogen, are exclusively (highly) metabolized in astrocytes and glutamine is only synthesized in astrocytes. Diffusion-weighted MRS has been suggested as a method that provides physiological and microstructural information by distinguishing those cell-specific metabolites based on diffusion effects. DW-MRS has been applied in the brain over the past 10 years, mainly to detect singlet resonances of NAA and creatine at clinical field strengths. With the use of UHF, DW-MRS data can be obtained on other metabolites of interest, such as Gln, NAAG, and Glu, due to improved spatial and (possibly) temporal resolution. However, so far, only five DW-MRS studies have been published in the human brain at 7T [67,151,152,153,154] by the same research group and very limited data is available on patient populations [154]. A functional DW-MRS was performed in the human brain at 7T to measure dynamic changes in diffusion of metabolites during brain activation in response to visual stimuli [151], indicating the importance of combining two modalities.

## 6. Cerebral Metabolism in Healthy State

With the development of sophisticated medical imaging techniques such as magnetic resonance spectroscopy (MRS), several research groups have investigated assessing cerebral metabolism in both healthy and diseased states.

### 6.1. Aging

The cerebral metabolism is known to be altered during the normal healthy aging process. There are four UHF-MRS studies [64,155,156,157] which have measured the metabolic alterations associated with healthy aging. A ^1^H-MRS study at 7T performed in 60 healthy volunteers [64] showed elevated levels of glia-related metabolites (tCr, Cho, MI) in the anterior cingulate cortex (ACC), hippocampus, and thalamus. Another study showed neuron-related metabolites (Glu, NAA) were lower in the occipital cortex and concluded with region-specific alterations of metabolite levels in elderly healthy volunteers [155].

### 6.2. Interventional Studies on Healthy Volunteers

A few interventional MRS studies at 7T have been performed on healthy volunteers to explore the effects of various treatments, such as acute stress [158], postexercise [36], gabapentin treatment [159] (drug for seizures and neuropathic pain), transcranial direct current stimulation (tDCS) [149], monocular eye deprivation [160], ebselen [161] (a drug for bipolar disorder), ketone supplements [105], hyperglycemia [86], and therapeutic use of psychedelic substances such as psilocybin [162]. Reduction in GABA levels were observed in the visual cortex after monocular eye deprivation [160], while gabapentin treatment increased GABA levels [159]. After tDCS, the GABA/Glu ratio increased, indicating the tDCS-induced neurotransmitter alterations in the auditory cortex of healthy volunteers [149]. Acute stress did not alter the concentrations of both Glu and GABA [158]. In contrast, acute ingestion of ketone supplements reduced both Glu and GABA levels [105]. The study with hyperinsulinemia observed a trend in hypothalamic GABA decrease in response to acute fall in glucose concentrations [86]. The substrate metabolism has been studied at 7T after postexercise, where lactate concentrations were increased without much changes in Glu and Gln, indicating the importance of alterative substrate to glucose during the recovery period [36]. An acute dose of ebselen reduced the inositol level and increased Glu, Gln, and GSH in the anterior cingulate cortex [161]. Acute effects of ecstasy with the psilocybin drug showed regional variations of Glu concentrations in the brain [162]. A difference in the intra- and extracellular pH map was observed between GM and WM on healthy volunteers using ^31^P-MRSI at 9.4T [140].

## 7. Altered Cerebral Metabolism in Various Brain Disorders

Although the cerebral metabolism is known to be altered in normal healthy conditions including aging, the same phenomenon is exacerbated in various brain disorders. It is important to note that such cerebral metabolic changes typically occur prior to noticeable structural abnormalities in the brain. Glucose hypometabolism is one of the most common characteristic features of several NDs that has been consistently reported in numerous PET studies.

### 7.1. Brain Tumors

So far, seven MRS studies at 7T [74,75,90,163,164,165,166] and two studies at 9.4T [140,167] have been performed in tumor patients. Among them, six studies [74,75,163,164,165,167] were focused on 2-hydroxyglutarte (HG) detection, as it is considered as a typical marker to distinguish IDH mutants of glioma in brain tumors. This is the very interesting ‘killer’ application of MRS in clinical research towards improving healthcare in the field of oncology. The 2-HG produced from alpha ketoglutarate (an intermediate product of TCA cycle, shown in Figure 3), and therefore the synthesis of glutamate and GABA, will be altered due to the lack of precursor molecule. Moreover, altered metabolic fluxes have been shown in tumor patients by applying ^2^H-MRS at 4T [56].

### 7.2. Neurodegenerative Diseases (ND_S_)

Alzheimer’s disease (AD) is one of the most common NDs, with a progressive cognitive decline and which involves the deposition of amyloid beta-plaques and tau tangles in several brain regions. Reduced NAA and increased myoinositol are consistently reported in AD patients and have been suggested as an early marker for diagnosis and AD progression. In addition to alterations in those high-abundant metabolites, two ^1^H-MRS at 7T showed alterations in low-abundant metabolites, such as increased ascorbic acid [71], in the parietal and occipital cortex, as well as decreased glutamate and GABA [82] in the posterior cingulate cortex (PCC), indicating a distinct neurochemical profile, especially in the cortex of AD patients. Moreover, two ^31^P-MRSI studies at 7T [168,169] revealed alterations in phosphate energy metabolites and membrane lipids, which are associated with decreased cognitive function.

Parkinson’s disease (PD) is a degenerative movement disorder caused by the selective neuronal loss of dopamine in the substantia nigra and development of inclusions called Lewy bodies. So far, only two ^1^H-MRS studies have been performed at 7T in PD patients [65,170]. In one study, elevated GABA concentrations were found in the pons and putamen of PD patients [65], while in another study, increased GSH concentrations were observed (in occipital cortex) in response to the infusion of N-acetylcysteine as a treatment for PD patients [170].

Amyotrophic lateral sclerosis (ALS) is a motor-neuron disorder characterized mainly by the irreversible loss of voluntary muscle function. Three ^1^H-MRS studies were performed at 7T in ALS patients, where reduced NAA and increased MI were reported in precentral gyrus [66], primary motor cortex, and pons [171]. Moreover, one of the studies at 7T demonstrated that those metabolic alterations were associated with function decline in ALS patients [172].

Multiple sclerosis (MS) is an autoimmune neurodegenerative disease which mainly involves neuroinflammation and demyelination. Although reduced tNAA has been consistently reported in earlier 3T MRS studies, a recent ^1^H-MRS study at 7T revealed the reduction in tNAA is purely due to NAA concentration and not by NAAG [173]. Moreover, 83% of lesions in MS patients showed an increase in the MI/NAA ratio, indicating the major role of astrogliosis in MS [174]. In a ^1^H-MRSI study at 7T, lower GSH levels were reported in the normal-appearing GM of MS patients, while GM is generally expected to have higher GSH concentrations than WM [175].

Epilepsy is a brain disorder that causes frequent unprovoked seizures. Two ^1^H-MRS studies [72,176] and two ^1^H-MRSI [177,178] studies have been performed at 7T in epilepsy patients. High GSH concentrations in PCC [72], as well as reduced NAA and Gln levels in hippocampus [176], were reported in epilepsy patients. A ^1^H-MRSI study at 7T introduced a new concept of “neurotransmitter networks” towards identifying spatial variations of neurotransmitter concentrations (e.g., Glu and GABA) and showed application in epilepsy patients [177].

Huntington’s disease (HD) is a neurodegenerative genetic disorder characterized by abnormal body movements and early cognitive decline that leads to dementia. So far, three ^1^H-MRS studies at 7T were performed in HD patients where reduced NAA was reported in the caudate nucleus, putamen [83], and PCC [179], while lower creatine levels were noted in the caudate nucleus and putamen [83]. In a longitudinal ^1^H-MRS study at 7T after 2 years follow-up of HD patients, MI and creatine were reduced in the caudate nucleus, while NAA and choline were reduced in putamen, indicating impaired energy metabolism and loss of neuronal integrity particularly in the deep brain structures of HD patients [180].

### 7.3. Psychiatric Disorders

Schizophrenia (SCZ) is a chronic mental disorder characterized by the loss of brain volume that leads to several psychological problems, including psychosis, hallucinations, abnormal motor behavior, and delusions. In total, fifteen ^1^H-MRS studies [38,63,70,181,182,183,184,185,186,187,188,189,190,191,192] were performed at 7T in patients with schizophrenia, mainly focused on the cortical metabolic abnormalities, and in one study, f-MRS [193] was performed in addition to proton MRS. Among them, eight studies [70,182,183,184,185,186,187,188] showed reduced Glu and Gln in the anterior cingulate cortex (ACC), while reduced GABA was reported in three studies [70,183,189], indicating altered glutamatergic and GABAergic activity. Moreover, elevated lactate was observed in ACC in two studies [38,70], indicating mitochondrial dysfunction in patients with schizophrenia. Interestingly a recent ^1^H-MRS study at 7T after 4 years follow-up of SCZ patients [181] indicates pronounced cortical metabolic abnormalities, such as reduced GABA, tcre, Cho, MI, and NAA in ACC of epilepsy patients, while GSH levels reduced gradually due to treatment effect.

Depression/major depressive disorder (MDD) causes a persistent feeling of sadness and loss of interest that can lead to various emotional and physical health problems. An f-MRS study at 7T revealed dynamic changes of reduced glutamate level during brain activation with Stroop task in patients with MDD [193].

Attention-deficit-hyperactive disorder (ADHD) is the most common brain disorder in children who typically show inattention as well as impulsive and hypermotoric behaviors. A ^1^H-MRS study at 7T in children with ADHD showed reduced GABA level in striatum [69], indicating poor inhibitory activity which could be targeted as a future treatment strategy for ADHD.

Migraine is a neurological disease typically characterized by moderate to severe throbbing headache with nausea which can last between 4 and 72 h. So far, only two ^1^H-MRS studies at 7T from the same research group were performed in patients with migraines, which showed increased glutamate in visual cortex [194] and decreased NAA in the cerebellum [195]. Among them, one study performed DW-MRS, where no changes in diffusion constants for glutamate were observed [194].

Anorexia nervosa (AN) is a serious mental illness characterized by fear of gaining overweight with strict measures to reduce required normal calorie intake that leads to high mortality and morbidity. Interestingly, a ^1^H-MRS study at 7T [196] showed reduced glutamate levels in the ACC, occipital cortex, and putamen, indicating poor excitatory activity in AN patients.

Drug addiction, such as the consumption of morphine or cannabis, is known to alter glucose utilization in the brain. A ^1^H-MRS study at 7T showed the reduction of striatal Glu concentrations on healthy occasional cannabis users [197], while another study from the same research group showed distinct metabolic profile associated with reward circuitry between acute and chronic cannabis users [198].

Additionally, 22q11.2 is a genetic disorder with the deletion of protein coding genes on chromosome 22 locus that expresses heterogeneous phenotypic expressions, including palatal anomalies, hypocalcemia, and congenital heart diseases. Three MRS studies at 7T on 22q11.2 patients has been performed from the same research group [199,200,201], and the authors observed the reduction of glutamate in the ACC and striatum, and showed the potential treatment benefits of riluzole on these patients.

## 8. Challenges and Future Perspectives

UHF-MRS is quite challenging, mainly due to the inhomogeneities of the field strength (B0), decreased receptive homogeneity (B1), stronger chemical shift displacement errors (CSDE), long T_1_ relaxation times (especially for proton), and high energy deposition. The development and availability of special MRS sequences (e.g., adiabatic pulses), a dedicated RF coil, and sophisticated postprocessing algorithms could resolve the major challenges of UHF. For instance, recently, an MRS consensus group recommended the use of the semiLASER sequence, which provides better localization, high SNR, minimal CSDE artifacts, and less susceptible for B0 and B1 inhomogeneities. Moreover, the high SNR achieved with semiLASER enables to perform MRS in deep structures of the brain to study the cerebral metabolism. Nowadays, several research groups have applied sLASER sequence to achieve good quality of MR spectra at UHF despite its challenges.

In addition to the overview of current MRS techniques to assess the cerebral metabolism, the potential metabolic insights obtained by these techniques at UHF in various brain disorders are summarized in this review (also provided in Supplementary Table S1). So far most of the UHF-MRS studies focused on the development of new techniques, MR sequences, shimming approaches on healthy volunteers, and very few studies available on patient populations. Among them, the majority of studies focused on schizophrenia, and earlier literature reviews indicated no UHF-MRS studies on post-traumatic stress disorder (PTSD) [202], autism spectrum disorder (ASD) [203], and stroke. Moreover, neurotransmission-related metabolites (Glu, Gln, GABA) have been commonly focused on UHF-MRS studies and limited data are available on brain substrate metabolism (e.g., BHB, glycogen and lactate), especially on patient populations. In several NDs, a shift from the oxidative to glycolytic pathway was observed, and it is plausible to suggest an important target for treatment strategies.

Besides the potential benefits of the X-nuclei MRS, the widespread use of X-nuclei applications is limited at UHF due to the requirement of a dedicated RF coil, which is generally custom-built by the researchers. Thus, X-nuclei applications are still in the development stage and researchers still need to work on required software, such as developing a postprocessing algorithm, robust automated procedures for shimming, and mathematical compartment model for the estimation of metabolic fluxes and hardware (e.g., RF coils, dipole pads for shimming) to further increase the spatial and temporal resolution. Although this review focused on outlining the current MRS methods to assess cerebral metabolism, in principle, these MRS methods can also be applied in other organs, such as the liver, skeletal muscle, and heart, to study different aspects of the metabolism.

## 9. Conclusions

MRS has immense potential and is the only noninvasive technique available to measure many metabolites in vivo in the cellular and regional levels in the human brain of both healthy and diseased states. With the high SNR and improved spectral resolution at UHF, MRS can provide reliable and reproducible measurements of neurochemicals in different regions of the brain and detect physiologically low-abundant metabolites (e.g., NAD, serine, and glycine) towards assessing the cerebral metabolism. In addition to ^1^H-MRS, X-nuclei, such as ^2^H, ^31^P, and ^13^C-MRS, at UHF can provide detailed metabolic information on a cellular level towards measuring metabolic fluxes in vivo. Moreover, UHF enables to acquire the fast mapping of different metabolite maps with a high anatomical specificity through ^1^H and ^31^P-MRSI and a high-resolution pH map by ^31^P-MRSI towards bringing the diagnostic potential of MRS in various brain disorders, especially for tumors. Interestingly, UHF has boosted the applications of advanced MRS, such as functional MRS and diffusion-weighted MRS, in terms of precision, reliability, and stability for longitudinal and dynamic measurements to monitor the aberrant metabolism in different (pathological) conditions of brain. Thus, multinuclear MRS at UHF has a great potential to measure cerebral metabolic changes accurately and has enabled the identification of potential biomarkers to diagnose, treat, and monitor the progression of several brain disorders.

## Figures and Tables

**Table 1:** MR characteristics of X-nuclei. Relaxation times of water in the human brain are presented for the nuclei ^1^H, ^2^H, and ^17^O. For ^13^C, relaxation times are denoted for liver glycogen from rat at 8.4T *. ^31^P- relaxation times are represented for PCr resonance in the human brain. An approximate chemical shift is represented for in vivo spectral range for each nucleus.

Nuclei	Spin	Natural Abundance (%)	Gyromagnetic Ratio (MHz/T)	Chemical Shift (ppm)	T_1_ at 7T (ms)	T_2_ at 7T (ms)	Common Observable Metabolites
^1^H	1/2	100	42.58	~6	~1600–2000 [23]	60–88 [24]	Glu, Gln, Asp, MI, Ins, Cre, NAA, NAAG, Cho, Tau
^2^H	1	0.016	6.54	~6	~350 [15]	20–30 [15]	Water, glucose, lactate, and Glx (Glu + Gln)
^17^O	5/2	0.037	−5.77	>500	5.46 [25]	4.32 [25]	Water
^13^C	1/2	1.1	10.71	>200	* 300 [26]	* 9–13 [26]	Glucose, glycogen, lactate, pyruvate, fatty acids, Glu, Gln, and Asp
^31^P	1/2	100	17.24	~30	3370 [14]	132 [14]	ATP, Pi, PCr, PME, PDE

**Table 2:** Overview of relevant metabolic substrates for cerebral metabolism. AD—Alzheimer’s disease; PD—Parkinson’s disease; MS—Multiple sclerosis.

Metabolic Substrates	Major Role	Human Brain Content	Where It Gets Metabolized/ Synthesized	Changes in Healthy State	Implications in Brain Disorders
Glucose [16]	Obligatory substrate for adult brain	1–2.5 mM [31]	All brain cells	Aging [32]	AD, PD, MS [16]
Lactate [33]	Maintain neuroenergetics and neuroplasticity	~1 mM [34]	All brain cells, synthesized in astrocytes and delivered to neurons	Stress [35], Exercise [36], Postnatal period, Hypoglycemia [37], Hypoxia [33] and Neuronal Activation [34]	Schizophrenia [38], Mitochondrial disorders [39], Tumors [40], Ischemic stroke [35] and Bipolar disorder [41]
Glycogen [42]	Cognition, memory and glutamateric neurotransmission	7.8 ± 0.3 µmol/g [43]	Mainly in Astrocytes	Hypoglycemia [44,45] and prolonged exercise [46]	Diabetes [47] and Lafora disease [48,49]
Ketone bodies [50]	Sustain cerebral metabolism briefly	0.25 mM [51]	Mainly in Astrocytes	Development period, neonates, Prolonged Hypoglycemia and starvation [50,52]	Brain injury, AD and PD [50,53]

## Supplementary Materials

The following supporting information can be downloaded at: https://www.mdpi.com/article/10.3390/metabo13040577/s1, Table S1: Comprehensive overview of all major brain diseases and corresponding cerebral metabolic alterations reported in UHF-MRS studies.
