# Supplementary material for: Multinuclear Magnetic Resonance Spectroscopy at Ultra-High-Field: Assessing Human Cerebral Metabolism in Healthy and Diseased States
Source: Metabolites. 2023 Apr 19;13(4):577. doi: 10.3390/metabo13040577 (PMC10143499; doi:10.3390/metabo13040577)
Supplement: Supplementary file 1 [file metabolites-13-00577-s001.zip › metabolites-2307624-supplementary.pdf]

Table S1. Comprehensive overview of all major brain diseases and corresponding cerebral metabolic alterations reported in UHF-MRS studies.

Author	Disease Type	Population (n)	Target Region	Techniques & B0	Main Parameters	Main Metabolic Alterations
Oeltzschner et al., 2019[82]	AD	13 controls & 13 MCI patients	ACC & PCC	^{1}H-MRS at 7T	STEAM, TR=3000 ms, TE=14 ms in ACC & 15 ms in PCC	GABA, Glu & NAA reduced in PCC while MI increased in ACC.
Marjanska et al., 2019[71]	AD	33 controls & 16 AD patients	PCC, OCC, WM	^{1}H-MRS at 7T	STEAM, TR/TE/TM=5000/8/32 ms	ASc increased in ACC & OCC, MI and tCho increased in PCC of AD patients.
Das et al., 2021[168]	AD	15 controls, 15 aMCI & 11 AD patients	Frontal, parietal, temporal & OCC	^{31}P-MRSI at 7T	FID, TR=0.5 s, delay=0.5 ms.	Mg^{2+} decreased in TL of AD, p-BEM decreased in aMCI.
Das et al., 2020[169]	AD	19 aMCI patients	Parieto-occipital lobe	^{31}P-MRSI at 7T	FID, TR=1-5 s, delay=0.5 ms,	higher BEM indices were associated with lower cognitive performance of memory in aMCI.
Emir et al., 2012[65]	PD	12 controls & 13 PD patients	Pons, putamen & substantia nigra	^{1}H-MRS at 7T	STEAM, TR/TE/TM=5000/8/32 ms	GABA increased in pons & putamen of PD.
Holmay et al., 2013[170]	PD	3 controls & 3 Gaucher & 3 PD disease	OCC	^{1}H-MRS at 7T	sLASER, TR/TE=5000/ 26 ms	N-acetylcysteine infusion increased glutathione levels in brain and blood.
Atassi et al., 2017[66]	ALS	12 controls & 13 ALS patients	Left precentral gyrus	^{1}H-MRS at 7T	STEAM, TR/TE/TM=5000/ 5/75 ms	tNAA, Glu & tCr reduced while MI increased in ALS patients.
Cheong et al., 2017[171]	ALS	17 controls & 19 ALS patients	Primary motor cortex & pons	^{1}H-MRS at 7T	sLASER, TR/TE=5000/ 26 ms	tNAA/MI ratio reduced in motor cortex & pons of ALS patients.
Cheong et al., 2018[172]	ALS	19 controls & 20 patients	Primary motor cortex & pons	^{1}H-MRS at 7T	sLASER, TR/TE=5000/ 26 ms	Neurochemical changes are associated with functional decline.
Gonen et al., 2020[72]	Epilepsy	10 controls, 19 TLE &16 IGE patients	PCC	^{1}H-MRS at 7T	STEAM, TR/TE= 8500/6 ms	GSH increased in PCC of IGE patients.
Pan et al., 2013[178]	Epilepsy	25 patients	Whole brain	2D & 3D ^{1}H-MRSI at 7T	Hahn SE, TR/TE=1500/ 40 ms	MRSI-determined abnormality and surgical resection region is related to outcome prediction.

Voets et al., 2017[176]	Epilepsy	12 controls, 11 TLE patients	Hippocampus	^{1}H-MRS at 7T	sLASER, TR/TE=6000/ 36 ms	Reduced Gln concentrations which correlated with impaired verbal memory performance.
Van Veenendaal et al., 2018[177]	Epilepsy	20 controls & 10 patients	Whole brain	^{1}H-MRSI at 7T	sLASER, TR/TE 5520/38 ms	Increased number of Glu and GABA connections (neurotransmitter network) in epilepsy patients.
Reid et al., 2018[182]	SCZ	26 controls & 23 patients	ACC	^{1}H-MRS at 7T	STEAM, TR/TE/TM = 10000/5/45 ms,	Glu & tNAA reduced in SCZ patients.
Rowland et al., 2016[38]	SCZ	29 controls & 27 patients	ACC	^{1}H-MRS at 7T	STEAM, TR/TE/TM = 3000/14/33 ms,	Lac increased in SCZ patients.
Thakkar et al., 2016[183]	SCZ	24 controls & 23 patients (first-degree) & 21patients with chronic SCH	Basal ganglia and OCC	^{1}H-MRS at 7T	sLASER, TR/TE= 5000/36 ms JDE-MEGAsLASER, TR/TE=5000/74 ms	Reduced GABA and Gln in OCC of SCZ patients.
Brandt et al., 2016[184]	SCZ	24 controls & 24 patients	ACC	^{1}H-MRS at 7T	STEAM, TR/TE/TM = 3000/14/28 ms,	Glu decreased in SCZ patients.
Godlewska etal., 2021[185]	SCZ	18 controls & 17 patients	ACC, DLPFC & putamen	^{1}H-MRS at 7T	STEAM, TR/TE= 5000/11ms,	Glu & Gln reduced in ACC but no change in DLPFC & putamen.
Kumar et al., 2020[186]	SCZ	45 controls & 28 patients	ACC, left insula & visual cortex	^{1}H-MRS at 7T	STEAM, TR/TE/TM = 200017/17 ms	Glu, Gln & GSH reduced in ACC
Limongi etl., 2021[190]	SCZ	20 controls & 19 patients	dACC, anterior insula (AI)	^{1}H-MRS at 7T	sLASER, TR/TE=7500/100 ms	Inhibitory activity within the dACC decreased with Glu levels whereas inhibitory activity in both the dACC and AI increased with GSH levels.
Marsman et al., 2014[189]	SCZ	23 controls & 17 patients	PFC, POC	^{1}H-MRS & GABA-edited MRS at 7T	sLASER, TR/TE=5000/28 ms, MEGAsLASER, TR/TE=4000/74 ms	GABA/Cr ratio reduced in SCZ patients.
Park et al., 2021[187]	SCZ	27 healthy & 47 FEB patients	dACC	^{1}H-MRS at 7T	sLASER, TR/TE=7500/100 ms	MRS Glu corelated with Receptor Specific Morphometric Signatures (RSMS)-NMDA receptor in FEB patients.

Wijtenburg etal., 2021[70]	SCZ	38 controls, 40 SCZ & 11 first-degree relatives	ACC, DLPFC, CSO, thalamus and hippocampus	^{1}H-MRS at 7T	STEAM, TR/TE/TM = 3,000/14/33ms	Multi-region differences in GABAergic and glutamatergic metabolites in SCZ, first-degree relatives and healthy controls.
Sandstrom et al., 2022[63]	SCZ	20 controls & 10 patients	ACC, DLPFC, thalamus, hippocampus, and the basal ganglia.	^{1}H-MRS at 7T	sLASER, TR/TE=3600/30 ms	A randomized clinical trial explored the potential benefits of Memantine (a drug to treat AD and psychosis).
Dempster et al., 2020[191]	SCZ	53 subjects	Dorsal anterior cingulate cortex	^{1}H-MRS at 7T	sLASER, TR/TE=7500/100 ms	No diff. in Glu & GSH levels between patients and controls. Glu is associated with severe functional impairment. Higher GSH is associated with shorter time to response.
Jeon et al., 2021[188]	SCZ	10 controls & 21 patients	Dorsal anterior cingulate cortex	^{1}H-MRS at 7T	sLASER, TR/TE=7500/100 ms	Glu reduced in patients and progressive level of Glu change is the best predictor to monitor treatment effect.
Wang et al., 2023[181]	SCZ	48 controls & 38 patients	ACC, CSO, OFR, thalamus, DLPFC	^{1}H-MRS at 7T	STEAM, TR/TE/TM=3000/14/33 ms	Glu reduced in both controls and patients in ACC. GABA, tcho, tcr, MI & NAA are reduced in ACC of patients. GSH nearly zero over time with treatment.
Wang et al., 2019[192]	SCZ	91 controls & 81 patients	ACC, CSO, DLPFC, OFR & thalamus	^{1}H-MRS at 7T	STEAM, TR/TE/TM= 3000/14/33 ms	NAA reduced but no change in NAAG. Lower levels of Glu, GSH, GABA in ACC.
Lind et al., 2020[64]	Aging	60 healthy volunteers, age from 18 to 79 years	Medial ACC, left DLFPC, left hippocampus & left thalamus	^{1}H-MRS at 7T	sLASER, TR/TE=3700/32 ms	MI increased in hippocampus & thalamus. tCho and Cre increased in ACC & hippocampus. Glu decreased in DLFPC.
Marsman et al., 2013[156]	Aging	33 volunteers, age from 18 to 31 years old	Medial frontal region	^{1}H-MRS at 7T	STEAM, TR/TE/TM= 2000/7/11 ms	Glu reduced in older individuals.
Schreiner et al., 2016[157]	Aging	30 LEM elderly subjects (70 years old)	posterior cingulate and precuneus (PCP)	^{1}H-MRSI at 7T	FID-TR=644 ms, acquisition delay= 2.5 ms	MI, Cho, Glu-Gln altered between GM & WM. High tNAA associated with low verbal learning & memory.
Marjanska et al., 2017[155]	Aging	17 young adults (19-22 years) &	PCC & ACC	^{1}H-MRS at 7T	STEAM, TR/TE/TM= 5000/8/32 ms	Asc, Glu and NAA reduced in ACC. tCre and MI increased in PCC.

		16 older adults (70-88 years)				
Tackley et al., 2021[173]	MS	11 MS and 4 AQP4Ab-NMOSD patients	NAWM, lesion	^{1}H-MRS at 7T	STEAM, TR/TE= 5000/11 ms	Not much differences in neurochemical profiles
Heckova et al., 2019[174]	MS	20 patients, 77 lesions	NAWM, lesion	^{1}H-MRSI at 7T	FID, delay=1.3 ms	83% of lesion shows increased MI/ NAA in MS patients.
Srinivasan et al., 2010[175]	MS	6 controls and 7 patients	GM, WM	^{1}H-MRSI at 7T	FID, TR=2000 ms	GSH is reduced in GM but not in WM.
Vingerhoets et al., 2019[201]	22q11.2	One patient	ACC & Striatum	^{1}H-MRS at 7T	STEAM, TR/TE/TM=5000/6 /10ms	Glu reduced in both ACC & striatum of 22q11.2 patients.
Vingerhoets et al., 2020[200]	22q11.2	17 patients & 20 controls	ACC & striatum	^{1}H-MRS at 7T	STEAM, TR/TE/TM=5000/6 /10ms	Riluzole decreased Glu in ACC of 22q11.2 patients.
Hooijdonk et al., 2022[199]	22q11.2	17 patients & 20 controls	ACC & striatum	^{1}H-MRS at 7T	STEAM, TR/TE/TM=5000/6/10ms	An association between glutamatergic functioning and brain volume in healthy controls but not in 22q11.2 patients.
Puts et al., 2020[69]	ADHD	24 controls & 26 with ADHD children	DLPFC, VMPFC, striatum, PMC	^{1}H-MRS at 7T	STEAM, TR/TE/TM=3000/14/26 ms,	GABA reduced in striatum of patients with ADHD
Van den Bogaard et al., 2011[83]	HD	44 subjects	caudate nucleus, putamen, thalamus, hypothalamus, and frontal lobe	^{1}H-MRS at 7T	STEAM, TR/TE/TM=2000/19/25 ms	NAA & Cre reduced in HD
Unschuld et al., 2012[179]	HD	12 controls & 12 patients	PCC	^{1}H-MRS at 7T	STEAM, TR/TE=3000/13 ms	NAA & Glu reduced in HD
Van den Bogaard et al., 2014[180]	HD	13 patients- 2 years follow up	caudate nucleus, putamen and PFC	^{1}H-MRS at 7T	STEAM, TR/TE/TM=2000/19/25 ms	Cre and MI reduced in caudate nucleus. NAA and Cho levels reduced in putamen of HD patients.

Zielman et al., 2014[195]	Migraine	19 controls & 18 patients	Cerebellum, OCC, pons & hypothalamus	^{1}H-MRS at 7T	STEAM, TR/TM/TE =2000/19/21 ms	Decreased tNAA/Cre ratio
Zielman et al., 2017[194]	Migraine	24 controls & 50 patients	Primary & secondary visual cortex	^{1}H-MRS& DW-MRS at 7T	sLASER, TR/TE=5000/30 ms. DW-MRS: PRESS, TR/TE=2000/120 ms	Glu increased in visual cortex
Godlewska etal., 2017[196]	Anorexia Nervosa	12 controls & 13 patients	ACC, OCC & putamen	^{1}H-MRS at 7T	STEAM, TR/TE=5000/11 ms	Glu reduced in all three voxels of AN patients. Inositol reduced in ACC & OCC.
Taylor et al., 2015[193]	MDD & SCZ	16 controls, 16 SCH, 16 MDD patients	ACC	^{1}H-MRS&f-MRS at 7T	STEAM, TR/TE/TM=3000/10/32 ms	Gln increase in SCZ during first run of task. Glu reduced in MDD during second run of task.
Emir et al., 2016[75]	Tumor	8 controls & 14 glioma patients	Tumor region	^{1}H-MRS at 7T	sLASER, TR=5000- 6000 ms, TE=110 ms	2-HG detectable and increased in tumor patients.
Ganji et al., 2017[163]	Tumor	12 patients	Tumor region	^{1}H-MRS at 7T	PRESS, TR/TE=2500/78 ms	Optimized PRESS at TE=78 enables to detect 2-HG in tumor patients.
Gruber et al., 2017[90]	Tumor	10 controls & 2 glioma patients	Frontal, Parietal & occipital cortex	^{1}H-MRSI at 3T&7T	FID-TR=600 ms, delay=1.5 ms	MRSI allow fast and reliable metabolite maps in approximately 6 minutes at 7 T than in approximately 30 minutes at 3 T.
Li et al., 2015[166]	Tumor	29 glioma patients	Tumor & healthy tissue	^{1}H-MRSI at 7T	SE, TR/TE=2000/30 ms	Gln, MI, glycine & GSH increased while tNAA reduced in tumor regions.
Verma et al., 2016[164]	Tumor	9 patients	Tumor region	2D L-COSY at 7T	2D-COSY sequence, TR/TE=2000/20 ms	2-HG detected in patients
Bisdas et al., 2016[167]	Tumor	18 patients	Tumor region	^{1}H-MRSI at 9.4T	STEAM, TR/TE/TM=2000/20/11 ms	Glu & Gln pool reduced and 2-HG detection in patients.
Berrington et al., 2017[74]	Tumor	9 patients	Tumor region	^{1}H-MRS at 3T & 7T	sLASER, TR/TE=5000-6000/110 ms	2HG detection and comparison at 3T &7T.

Shen et al., 2019[165]	Tumor	20 patients	Tumor region	^{1}H-MRS at 7T	sLASER, TR/TE=5000-6000/110 ms	IDH2 mutants have higher 2HG and MI.
Mason et al., 2019[197]	Drug addiction	20 healthy occasional cannabis users	ACC & striatum	^{1}H-MRS at 7T	STEAM, TR/TE/TM=5000/6/10 ms	Glu increased in striatum after an acute dose of cannabis.
Mason et al., 2021[198]	Drug addiction	12 occasional & 12 cannabis users	ACC & Striatum	^{1}H-MRS at 7T	STEAM, TR/TE/TM=5000/6/10 ms	In occasional users, acute dose of cannabis increased Glu in striatum and associated with reward circuitry but such changes absent in chronic users.

Supplementary Table S1: Comprehensive overview of all major brain diseases and corresponding cerebral metabolic alterations reported in UHF-MRS studies. ACC-anterior cingulate cortex, PCC-posterior cingulate cortex, MCI-mild cognitive impairment, OCC-occipital cortex, TLE-temporal lobe epilepsy, DLFPC- dorsolateral prefrontal cortex, POC-parieto-occipital cortex, FEP-first-episode psychosis, CSO- centrum semiovale, OFR- Orbitofrontal region, VMPFC- ventromedial prefrontal cortex, MDD- major depressive disorder, LEM- low episodic memory, FID-free induction decay, TL- temporal lobe, p-BEM- phosphate brain energy metabolism, SCZ-Schizophrenia, NMDA- N-methyl D-aspartate. NAWM- normal appearing grey matter.
